# Classification and Identification of Contaminants in Recyclable Containers Based on a Recursive Feature Elimination-Light Gradient Boosting Machine Algorithm Using an Electronic Nose

**DOI:** 10.3390/mi14112047

**Published:** 2023-10-31

**Authors:** Fushuai Ba, Peng Peng, Yafei Zhang, Yongli Zhao

**Affiliations:** School of Mechanical and Automotive Engineering, Shanghai University of Engineering Science, Shanghai 201620, China

**Keywords:** electronic nose, contaminant classification, recursive feature elimination, light gradient boosting machine

## Abstract

Establishing an excellent recycling mechanism for containers is of great importance for environmental protection, so many technical approaches applied during the whole recycling stage have become popular research issues. Among them, classification is considered a key step, but this work is mostly achieved manually in practical applications. Due to the influence of human subjectivity, the classification accuracy often varies significantly. In order to overcome this shortcoming, this paper proposes an identification method based on a Recursive Feature Elimination-Light Gradient Boosting Machine (RFE-LightGBM) algorithm using electronic nose. Firstly, odor features were extracted, and feature datasets were then constructed based on the response data of the electronic nose to the detected gases. Afterwards, a principal component analysis (PCA) and the RFE-LightGBM algorithm were applied to reduce the dimensionality of the feature datasets, and the differences between these two methods were analyzed, respectively. Finally, the differences in the classification accuracies on the three datasets (the original feature dataset, PCA dimensionality reduction dataset, and RFE-LightGBM dimensionality reduction dataset) were discussed. The results showed that the highest classification accuracy of 95% could be obtained by using the RFE-LightGBM algorithm in the classification stage of recyclable containers, compared to the original feature dataset (88.38%) and PCA dimensionality reduction dataset (92.02%).

## 1. Introduction

The recycling of containers can not only effectively decrease the disposal pressure of waste and reduce environmental pollution, but can also provide a large number of job positions [1]. The recycling of containers involves a series of steps such as classification and identification, cleaning, drying, shredding, and regeneration. Of all these steps, classification is the most important [2,3], because recycling value will be effectively increased by classifying waste containers. However, many factors such as the container’s size [4], color [5], pose [6], shape [7], external damage [8], internal contamination [9], and material [10] make achieving highly accurate classification and identification very challenging.

At present, the classification and identification of containers are mainly carried out manually, and have the limitations of high cost and low efficiency [11,12]. Additionally, some residual toxic and harmful gases may exist in those containers, which could hurt human health [13,14]. In addition, the subjectivity of the inspector can lead to inconsistent results [15,16]. To solve the above-mentioned problems, research into identification methods based on intelligent devices has become a popular research domain in recent decades. For example, Wang et al. [17] classified plastic bottles with different position relationships and colors based on image recognition, but detection inside the containers was not considered, and the experimental platform required strict lighting conditions. Dimitris et al. [18] used an online cloud computing platform with a distributed architecture for solid waste classification; therefore, the response speed limited its application for fast detection. Zhang et al. [19] proposed a recyclable waste classification model based on the combination of image classification and deep learning, and confirmed that this model could improve classification results on the TrashNet dataset; however, this model required a very large amount of clear image data for training, and its reliability was unstable. Wang et al. [20] proposed an innovative design concept of a smart recycling system based on Extenics to solve conflicts in cosmetic container recycling, but different categories of cosmetic containers were not investigated in this study.

Using an electronic nose system is a promising approach to solving the problems of classification and identification of contaminants. Actually, electronic noses have been proven to be effective for the classification and identification of contaminant gases. For instance, Wen et al. [21] detected the odors of rotten fruits with the help of an electronic nose, and achieved efficient identification of fruit freshness. Savirio et al. [22] applied an electronic nose to pre-adhesive recognition of relevant pollutants on the surface of composite fiber-reinforced polymers (CFRP). Herrero et al. [23] used an electronic nose to classify and quantify different pollutant gases in the air. Zhang et al. [24] identified six indoor air pollutants (formaldehyde, benzene, toluene, carbon monoxide, ammonia, and nitrogen dioxide) as air quality indicators, and classified the data collected using an electronic nose. Liu et al. [25] proposed a non-destructive method for detecting peach fungal contamination using an electronic nose, and showed that the electronic nose has high discrimination accuracy. Mesías et al. [26] also used an electronic nose as the predictive tool for detecting the chemical pollutants in roasted almonds.

In this paper, a model based on the Recursive Feature Elimination-Light Gradient Boosting Machine (RFE-LightGBM) algorithm is proposed in the classification stage of contaminants for recyclable containers. Based on the experimental results of using the proposed model, the difference in the classification accuracies on three datasets (the original feature dataset, principal component analysis (PCA) dimensionality reduction dataset, and RFE-LightGBM dimensionality reduction dataset) was firstly investigated. Subsequently, the PCA method and RFE-LightGBM algorithm were applied to reduce the dimensionality of the feature dataset, and the differences between the two methods were analyzed, respectively. Finally, the classification accuracies on these three datasets were discussed as well.

## 2. Algorithm Theory

**Figure 1:**
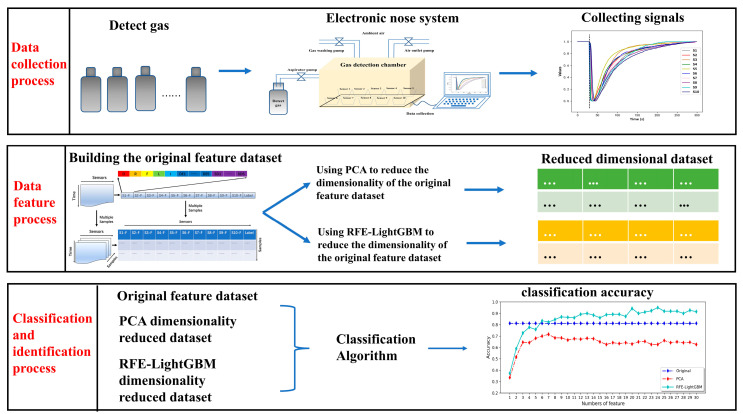
Flowchart of the contaminant classification approach based on the RFE-LightGBM algorithm using electronic nose.

Figure 1 shows the flowchart of the contaminant classification model proposed in this paper, which mainly consists of three processes: the data collection process, data feature process, and classification and identification process.

### 2.1. Light Gradient Boosting Machine (LightGBM)

The main idea of the optimal feature splitting point in the LightGBM algorithm [27,28] is as follows:

Assuming a dataset containing M samples and N features is given, LightGBM is an integrated model composed of *K* basic models, where each basic model represents a tree (representing different categories). Therefore, the predicted output of the integrated model can be expressed as Formular (1):

(1) $${\hat{y}}_{i}=\phi (x_{i})=\sum_{K=1}^{K}f_{k}(x_{i})$$

where $x_{i}$ is the characteristic value of the gas sample, $f_{k}$ is the predicted value of the *K*-th tree, and ${\hat{y}}_{i}$ represents the current predicted value. Equation (1) represents the sum of the predicted values of *K* regression trees (the weights of the leaf nodes divided according to the corresponding decision rules of the regression tree) given an input $x_{i}$. By iterating on each prediction tree and fitting the current difference to obtain the optimal model, we define the objective function as Formular (2):

(2) $$\mathrm{target}=\sum_{i=1}^{m}l({\hat{y}}_{i},y_{i})+\sum_{K=1}^{K}\Omega (f_{k})$$

where $l({\hat{y}}_{i},y_{i})$ is the loss function between the predicted value and the actual value. $\Omega (f_{k})$ represents the penalty term for the complexity of the model to balance the complexity of the model, and can be determined using Formular (3):

(3) $$\Omega (f_{k})=\mu T+\frac{1}{2}\lambda {\left\|\omega \right\|}^{2}$$

where *μ* and *λ* represent the penalty coefficient, *T* represents the number of leaf nodes for a given tree, and ||*ω*||^2^ is the square of the number of nodes on each leaf (predicted to be of the same category). When training the *K*-th tree, the first two *K*-1 trees in the front are known, and the unknown is the *K*-th tree. That is, based on the known decision tree constructed earlier, the *K*-th tree is constructed, and the predicted value of the *K*-th tree is represented by Formular (4):

(4) $${\hat{y}}_{i}^{\left(k\right)}={\hat{y}}_{i}^{\left(k-1\right)}+f_{k}(x_{i})$$

Taking Formular (4) into Formular (2), Formular (5) can represent the new objective function obtained:

(5) $$\mathrm{target}=\sum_{i=1}^{m}l({\hat{y}}_{i}^{\left(k-1\right)}+f_{k}(x_{i}),y_{i})+\Omega (f_{k})+C$$

Then the second-order Taylor approximation formular can be used to expand the objective function, as shown in Formular (6):

(6) $$\mathrm{target}=\sum_{i=1}^{m}\left[l({\hat{y}}_{i}^{\left(k-1\right)},{\hat{y}}_{i})+g_{i}f_{k}(x_{i})+\frac{1}{2}h_{i}f_{k}^{2}(x_{i})\right]+\Omega (f_{k})+C$$

where $g_{i}=\partial {\hat{y}}^{\left(k-1\right)}{\left({\hat{y}}^{\left(k-1\right)}-y_{i}\right)}^{2},h_{i}=\partial^{2}{\hat{y}}^{\left(k-1\right)}{\left({\hat{y}}^{\left(k-1\right)}-y_{i}\right)}^{2}$ in Formular (6) is the first derivative and the second derivative of the Loss function, respectively; the best classification characteristics are determined using Formular (7).

(7) $$\omega_{j}=-\frac{G_{j}}{H_{j}+\lambda }$$

$G_{j}=\sum_{i\in I_{j}}g_{i},H_{j}=\sum_{i\in I_{j}}h_{i}$ The final objective function can be represented as follows:

(8) $$\mathrm{target}=-\frac{1}{2}\sum_{j=1}^{T}\frac{G_{j}^{2}}{H_{j}+\lambda }+\mu T$$

Finally, the information gain of all features is determined according to Formular (9):

(9) $$Gain=\frac{1}{2}\left[\frac{G_{L}^{2}}{H_{L}+\lambda }+\frac{G_{R}^{2}}{H_{R}+\lambda }-\frac{{\left(G_{L}+G_{R}\right)}^{2}}{H_{L}+H_{R}+\lambda }\right]-\mu$$

Among them, *G_L_* + *G_R_* = *H_L_* + *H_R_* is the left and right branch sample set, split based on the best feature. The larger the value of the gain, the more it can reduce the loss of the objective function after splitting. This method can sort features based on the ranking of information gain and select the feature with the highest gain as the optimal splitting point.

### 2.2. Recursive Feature Elimination-Light Gradient Boosting Machine (RFE-LightGBM) Feature Selection Algorithm

**Figure 2:**
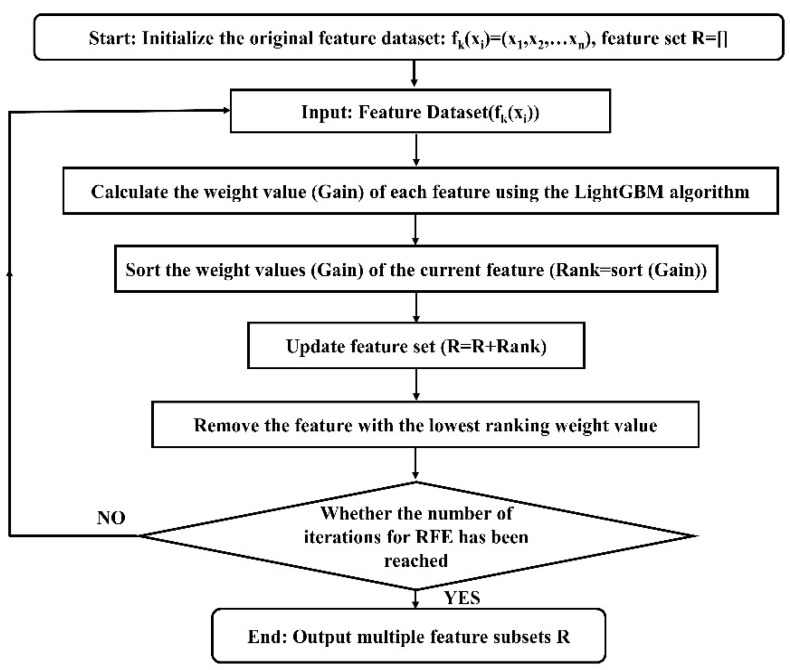
Flowchart of the data feature process based on the RFE-LightGBM algorithm.

LightGBM was used as the base model for the feature recursive elimination algorithm to model the original dataset in this study. After the corresponding weight values were calculated, all features could be sorted according to their weight values. Then, the features with the lowest weight values were successively deleted from the feature dataset using recursive feature elimination (RFE) [29,30,31,32], and the features were iterated circularly (the iteration number is equal to the dimension of the original feature dataset). Finally, sorting tables related to the multiple feature weight values could be obtained. The feature selection algorithm based on RFE-LightGBM is shown in Figure 2.

### 2.3. Algorithm Evaluation Criteria

In order to obtain the optimal feature subset, the classification accuracy (ACC = (TP + TN)/(TP + TN + FP + FN)) [33] was used to evaluate the score of each feature subset. The feature subset with the highest accuracy score was chosen as the best feature dataset to verify the classification results of the test data obtained based on the proposed model.

## 3. Materials and Methods

### 3.1. Electronic Nose System

**Figure 3:**
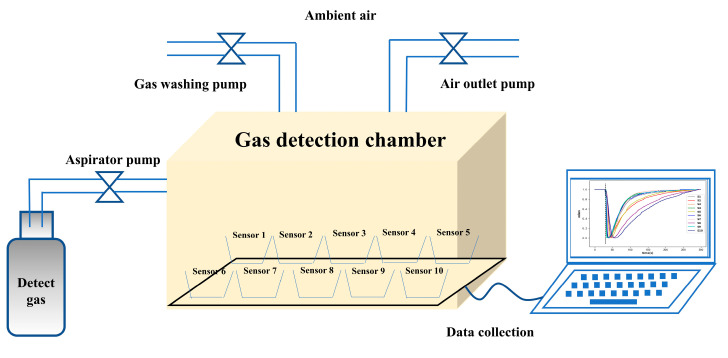
Schematic of the electronic nose system used in the current work.

Figure 3 shows the schematic of the electronic nose system used in this work, which consists of three components: a gas sensing array (10 homemade MEMS metal oxide sensors; because different sensors have different sensitivities to different gases, it is better to choose sensors with high sensitivity for different practical applications) [34,35]; a gas collection module; and a data acquisition module. The sensor parameters of the gas sensing array are listed in Table 1. The gas collection module is made of a 2L PE box and equipped with three pumps (the flow rate is approximately 800 mL/min). During the whole process of the experiment, the equipment, including the pumps, is controlled by a computer. The data acquisition module collects the response signal of the electronic nose to the detected gas and transfers it to the computer.

### 3.2. Experimental Procedures

In this work, barreled water buckets were used as recyclable containers. Under relatively fixed temperature and humidity conditions, the electronic nose system built in 3.1 was used to classify and identify contaminant gases in the recyclable container. 

For 5 consecutive days, residual gases (cigarette butts, coffee, liquor, and vinegar) in 3 concentration levels (10%, 30%, 50%), and uncontaminated barrels (100%) were classified into 13 categories. Each category of gas was detected 20 times each day (all the substances to be detected were poured out from the containers before measurement), so that a total of 100 data were obtained for each category of gas sample. The contaminant (taking coffee as an example, representing the ratio of the coffee volume to the whole volume of a barreled water bucket) and the percentage concentration of the experimental sample (e.g., a concentration of 50% means the volume ratio of contaminant in the recyclable container before measurement, which is expressed as the gas concentration in the recyclable container) are listed in Table 2. Details of the experimental procedure are as follows:

(1) The sensing array was preheated for 30 s to bring the baseline sensor resistance values to a steady state.
(2) The aspirator pump was turned on at the 30 s mark, and sent the gas to the detection chamber. The response signal of the sensor to the gas during the pumping time was collected.
(3) The aspirator pump was turned off at the 35 s mark, and the gas washing pump and air outlet pump were turned on (purging the gas detection chamber with ambient air) until all sensor resistance values returned to the original baseline values.
(4) Repeat the operations of steps 1–3 until data collection is completed for all target detectors.

## 4. Results

### 4.1. Response Curve of the Electronic Nose System

**Figure 4:**
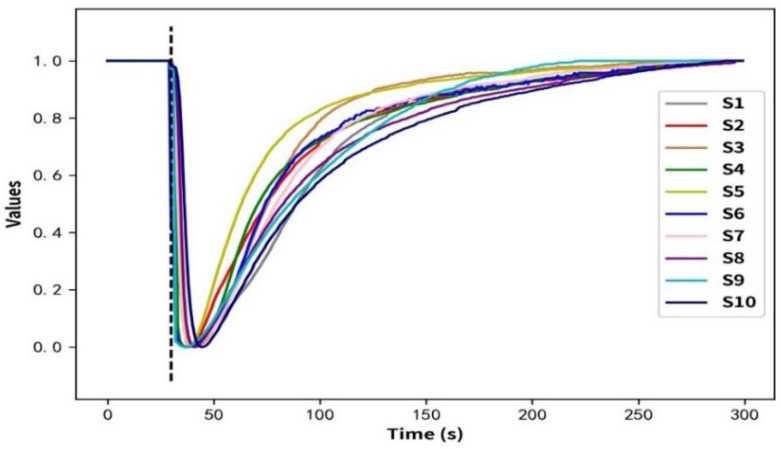
Curves of the original response data, processed according to Formular (10) for sample G0.

Affected by different sensitive materials, sensors will have different response sensitivities to the same target gas [36,37,38]. Moreover, environmental factors could also cause the baseline fluctuation of the sensors [39,40]. In order to eliminate these effects, we processed the collected signals according to the following formular:

(10) $$values=\frac{x-\min(x)}{\max(x)-\min(x)}$$

where *x*, min (*x*), and max (*x*) represent all the original data of each sensor, the minimum, and the maximum value in the data, respectively. Figure 4 shows the result curve of the raw data processed according to Formular (10). The horizontal axis represents the detection time, and the vertical axis represents the value after the change in the original data. It can be observed that the sensor array reached a stable state during the preheating phase (0–30 s). When the gas enters the detection chamber (30 s: black dashed line), the resistance values of the sensor array will decrease with the increase in the concentration of the gas. After stopping the gas supply, the resistance values of the sensor array return to the initial steady state with time.

### 4.2. Feature Datasets Constructed by Manual Methods

**Figure 5:**
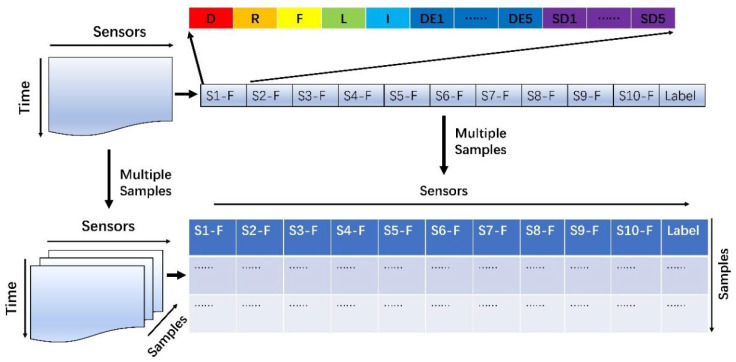
Schematic of the original feature dataset construction.

In this study, we extracted four steady-state features and three transient features from the pre-processed signals of each sensor [41], with a total of 15 features (1 + 1 + 1 + 1 + 1 + 1 + 5 + 5 = 15). The features extracted from all sensors (10 in total) are represented by different colors, and all the features are connected in order to form a feature vector that includes 150 features (10 × 15 = 150). Additionally, all the sample feature vectors are stacked together to form a feature dataset, as shown in Figure 5. The data collected by each sensor during the pumping period were defined as x^i^, and their detailed description is shown in the formular in Table 3 (where $x_{\max}^{i},x_{\min}^{i}$ represent the maximum value and minimum value).

### 4.3. Dimensionality Reduction of Feature Datasets

The feature dataset constructed using the above method has high dimensionality (with each feature vector containing 150-dimensional features). In order to speed up the model training, pre-processing of the feature dataset is necessary for eliminating the redundant features before training.

**Figure 6:**
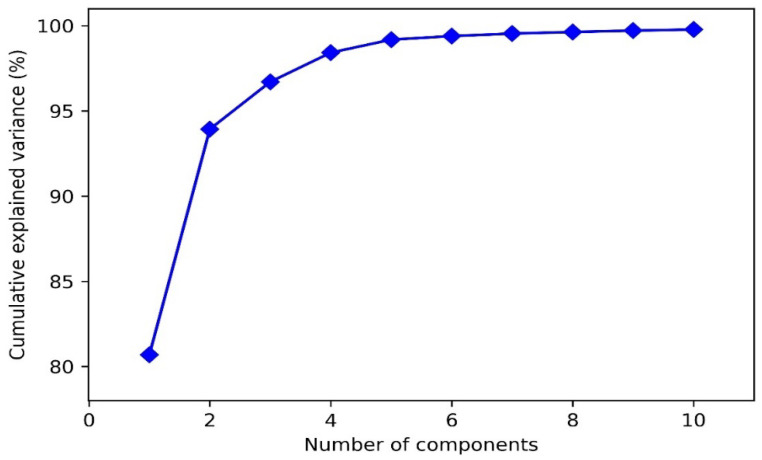
Cumulative variance of the principal components of the original feature dataset through PCA analysis.

In the field of gas identification, principal component analysis (PCA) is a widely used method [42]. Figure 6 shows the variance contribution of each principal component calculated using the PCA method, and the cumulative variance contribution of the first two and three principal components is 93.93% and 96.71%, respectively. When considering the first 10 principal components, the cumulative variance contribution could reach up to 99.78%.

**Figure 7:**
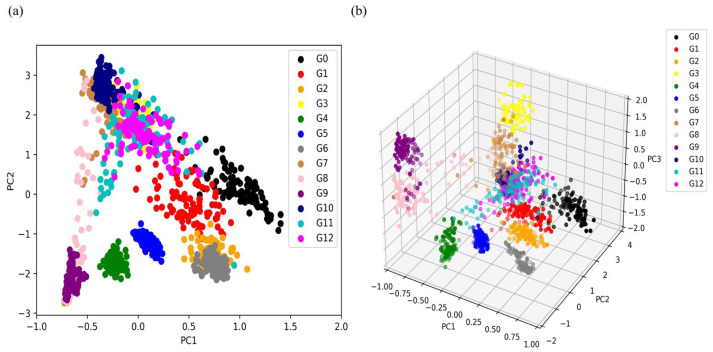
Visualization distribution of PCA analysis: (**a**) scatterplot of the first two principal components; (**b**) scatterplot of the first three principal components.

Figure 7 shows the visualization results of the original feature dataset after dimensionality reduction using PCA. It can be observed that when only the first two principal components are focused, there is a large amount of overlap between different categories, indicating that PCA cannot classify well (Figure 7a). Figure 7b shows the sample visualization distribution when applying the first three principal components. Here, the displayed classification effect is more obvious. It can also be seen that the ability to distinguish different samples has improved, but there are still some overlapping samples.

**Figure 8:**
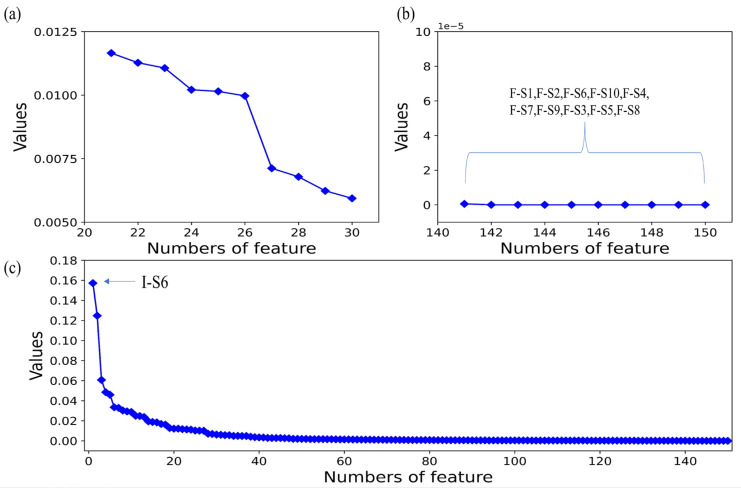
Information gain calculated according to the RFE-LightGBM method: (**a**) features with an information gain weight value higher than 0.01; (**b**) features with an information gain weight value of zero; (**c**) percentage of information gain weight value for all features.

Figure 8c shows the percentile weight values obtained using the RFE-LightGBM method for the features. The feature with the highest score is I-S6 (which is the integral feature of the sixth sensor), which means this feature provides the greatest information gain in classification. In contrast, as shown in Figure 8b, all the fractional difference feature contribution values were zero. This means that this type of feature is completely unhelpful for the classification. In addition, it can be seen in Figure 8a that only 26 features have contribution values higher than 0.01. Therefore, in order to reduce the dimensionality of the feature dataset, features with relatively low contribution rates could be discarded.

Table 4 shows 26 features with weight values greater than 0.01, with a total of 0.8442. Among them, integral features and derivative features made up a high proportion (17/26), which also indicates they make high contributions to gas identification. This shows that the integration features and derivative features can accurately express the information of the original data. Integral features can provide the best features, and derivative features can explain the rate and acceleration of the reaction. At the same time, the number of features formed by sensors S6, S7, and S8 correspond to 16/26. This shows that these features may have a significant impact on the classification and identification results. Therefore, the result confirms that the RFE-LightGBM method can utilize the original data information preserved in a small number of features in the model.

**Figure 9:**
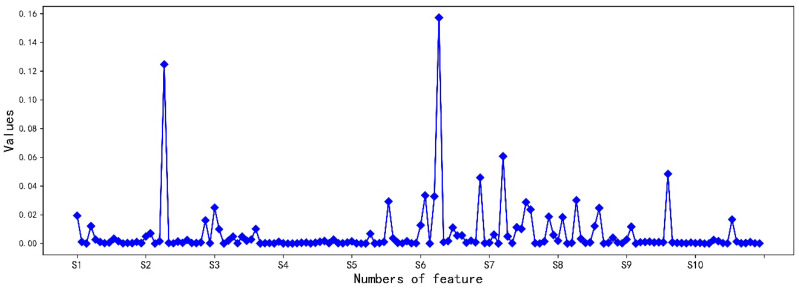
Percentages of feature information gain weight values for each sensor.

Figure 9 shows the importance score of each feature on different sensors. The larger values represent greater contributions to gas classification and identification. In addition, it can be seen that the feature weight of S4 is relatively low, which also indicates that S4 has a relatively small impact on the identification result. Therefore, removing S4 may not only reduce the amount of data generated and shorten the time for feature preprocessing, but also further reduce the power consumption of the sensor. In summary, the application of RFE-LightGBM in the field of gas identification not only effectively optimizes the sensing array, but also reduces the dimensionality of the feature dataset, thereby utilizing a small number of features to retain a large amount of original information.

**Figure 10:**
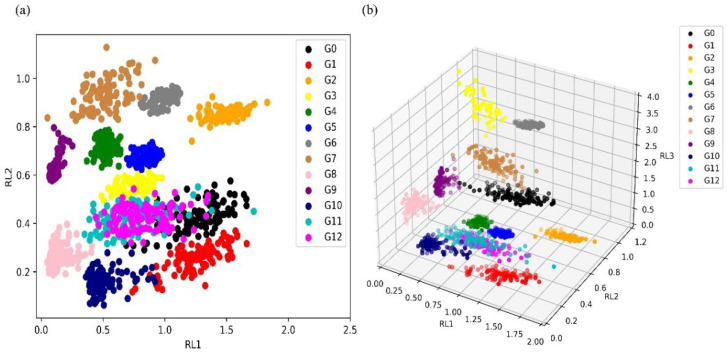
Visual distribution of samples after feature selection using the RFE-LightGBM method: (**a**) scatterplot of the first two principal components; (**b**) scatterplot of the first three principal components.

The visualized distribution of samples obtained using the RFE-LightGBM feature selection method was shown in Figure 10. The distribution of samples in different categories using the first two features is shown in Figure 10a. Although there are still overlapping phenomena between different categories, it is obviously lower than PCA. The visualization using the first three features also yielded the same conclusion (Figure 10b). After using the RFE-LightGBM method for feature selection, the distinction between different samples is more obvious.

## 5. Discussion

In this paper, a BPNN (back propagation neural network) model with one hidden layer [43] was constructed as a classifier. The number of input layers was equal to features, the number of output layers was equal to categories, and the categories were labeled with one-hot coding. The number of neurons in the hidden layer was defined as 10, and sigmoid was used as the activation function. To prevent the overfitting of the model, a five-fold cross-validation was used in the training process.

**Figure 11:**
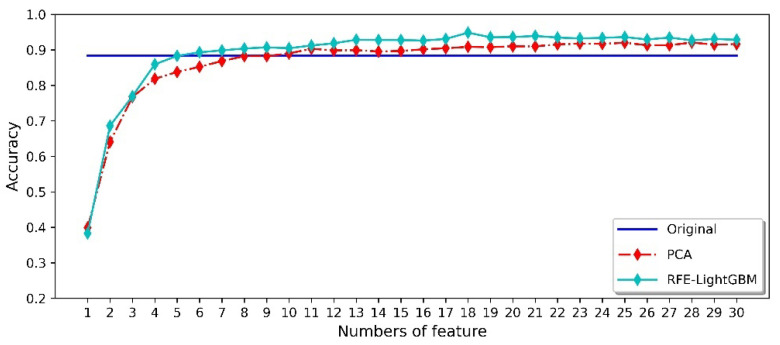
Classification accuracies of the different methods for randomly divided datasets.

In order to compare the influence of different feature processing methods on the final classification results, 80% of the data from three datasets (randomly divided among the original feature dataset, PCA dimensionality reduction dataset, and RFE-LightGBM dimensionality reduction dataset) were used for model training. The remaining data were used for model validation, and the results obtained are shown in Figure 11. The original feature dataset (1-150 dimensional features) obtained an average classification and identification accuracy of 88.38%. At the same time, one can also see that most PCA methods achieved lower accuracy than the RFE-LightGBM method. Compared with the PCA method, the RFE-LightGBM method can not only reduce the dimensions of the original feature dataset, but also obtain 94.84% classification accuracy using the first 18 features.

When performing gas identification, odors can change over time, which in turn is reflected by differences in the collected data. For example, Mahdi et al. [44] classified a variety of cheeses with different storage periods, Huang et al. [45] used RBF-ANN to assess fish freshness, and Madiha et al. [46] applied an electronic nose system for determining milk storage dates. The above studies have proved that the gas data collected on different days will affect the identification results. Therefore, when the electronic nose system is used to detect recyclable containers, the storage time of the contaminants in the containers will interfere with the final classification accuracy. If time interference can be overcome, the misclassification rate can be effectively reduced, and thus the robustness of the classification model can be significantly improved. Consequently, to explore the effect of gas data collected on different days on the classification and identification results, we divided the data into five schemes. We used different days of data as the training dataset and unknown days of data as the testing dataset, as shown in Table 5.

**Figure 12:**
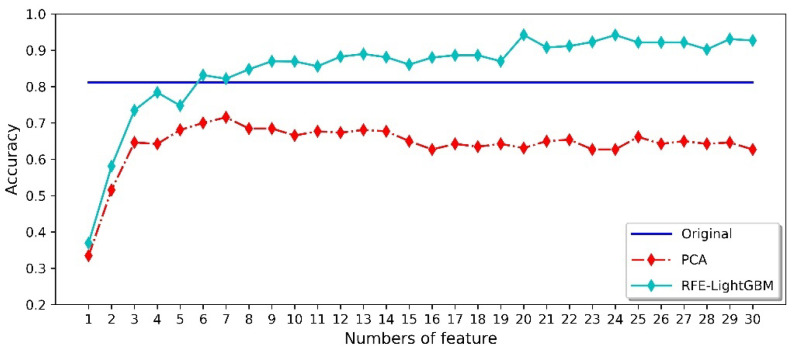
The classification accuracy of the Scheme 1 dataset based on different methods.

The training and testing datasets were divided into five datasets (see Table 5) to compare the effect of different feature preprocessing methods on the final classification results of each scheme. Figure 12 shows the recognition accuracy of the Scheme 1 dataset. We used the original feature dataset of 1-150 dimensions to train the model, and obtained an average accuracy of 81.15%. Meanwhile, we observed that most PCA methods had lower recognition accuracy than that of the average of the original feature dataset. PCA is an efficient dimensionality reduction method, and can reduce the computational complexity of the model. However, the traditional PCA method in the field of gas identification is not a good way to classify the samples. The RFE-LightGBM method can not only reduce the dimension of the feature dataset, but can also significantly improve the final classification effect. When the first 20 features were used for model training, the highest verification accuracy reached 94.23%. In addition, it can be observed in Figure 11 and Figure 12 that the average classification accuracy of the 1-150-dimensional original feature dataset decreased. The classification accuracy obtained using the PCA method is significantly reduced, indicating that the gas data collected on different days have different principal components. Moreover, the RFE-LightGBM method still shows good classification accuracy. Therefore, the application of the RFE-LightGBM method for feature selection can overcome the impact of odor changes over time.

Table 6 shows the classification accuracy of various data-partitioning schemes under different methods. It can be observed that the dimensionality of the feature dataset can be reduced via the PCA method, but the classification accuracy of the validation dataset also decreases significantly. In contrast, the application of the RFE-LightGBM method can not only reduce the dimensionality of the feature dataset, but also improve the classification accuracy. Even if the validated gas data come from different days, our built model achieves the best performance and also shows good classification and identification ability, where the highest validation accuracy result reaches 95.00%.

## 6. Conclusions

In this paper, an electronic nose system using the RFE-LightGBM algorithm was employed to classify and identify the contaminants in recyclable containers. The main results are as follows:

i. The use of electronic nose systems in the classification and identification of recyclable containers can compensate for the shortcomings of manual and other intelligent devices.
ii. Compared with PCA, RFE-LightGBM is an effective feature extraction method. It can not only reduce the dimensionality of the feature dataset, but also improve the classification accuracy.
iii. Using the RFE-LightGBM method in gas classification can overcome the influence of odor change over time. The highest classification accuracy reaches 95%.

## Figures and Tables

**Table 1:** Characteristics of the employed sensors in the electronic nose system.

Sensor	Main Test Objects	Detection Range (ppm)	Response Time (s)
S1	Ethanol, Acetone, Hydrogen Sulfide	0.1–500	<20
S2	VOCs, Smog	1–500	<10
S3	Ethanol, Hydrogen Sulfide, Acetone	1–500	<20
S4	Hydrogen	0.1–300	<10
S5	Hydrogen Sulfide	0.5–300	<20
S6	Ammonia	10–300	<10
S7	Ethanol	1–500	<20
S8	VOCs	10–500	<20
S9	Hydrogen Sulfide, Carbon Monoxide	1–500	<10
S10	Acetone, Hydrogen Sulfide	0.1–500	<10

**Table 2:** The composition and concentration of experimental sample gases.

Sample Label	Contaminant	Gas percentage Concentration
G0	Water	100%
G1	Cigarette	10%
G2	Cigarette	30%
G3	Cigarette	50%
G4	Coffee	10%
G5	Coffee	30%
G6	Coffee	50%
G7	Liquor	10%
G8	Liquor	30%
G9	Liquor	50%
G10	Vinegar	10%
G11	Vinegar	30%
G12	Vinegar	50%

**Table 3:** Gas features extracted using manual methods.

Symbol Mark	Number	Feature Description	Function
D	1	Difference	$x_{\max}^{i}-x_{\min}^{i}$
R	1	Relative difference	$x_{\max}^{i}/x_{\min}^{i}$
F	1	Fractional difference	$\left(x_{\max}^{i}-x_{\min}^{i}\right)/x_{\min}^{i}$
L	1	Logarithm difference	$\log\left(x_{\max}^{i}/x_{\min}^{i}\right)$
I	1	Integral	$\int_{0}^{5}x^{i}(t)dt$
DE	5	Derivative	$dx^{i}(t)/dt$
SD	5	Second derivative	$d^{2}x^{i}(t)/dt^{2}$

**Table 4:** Weight value details of the first 26 obtained features higher than 0.01, obtained using RFE-LightGBM analysis.

Feature Name	Importance
I-S6	0.1571
I-S2	0.1246
L-S7	0.0607
DE5-S9	0.0484
SD4-S6	0.0458
R-S6	0.0334
L-S6	0.0327
I-S8	0.0301
DE4-S5	0.0292
DE4-S7	0.0287
D-S3	0.0250
DE5-S8	0.0247
DE5-S7	0.0236
D-S1	0.0194
SD4-S7	0.0187
R-S8	0.0183
DE4-S10	0.0167
SD4-S2	0.0161
D-S6	0.0127
L-S1	0.0121
DE4-S8	0.0121
R-S9	0.0116
DE2-S7	0.0112
DE3-S6	0.0110
DE5-S3	0.0102
DE3-S7	0.0101
SUM	0.8442

**Table 5:** The datasets constructed according to the time of acquisition.

Datasets	Number of Days of Training Data Collection	Number of Days of Testing Data Collection
Scheme 1	2-3-4-5	1
Scheme 2	1-3-4-5	2
Scheme 3	1-2-4-5	3
Scheme 4	1-2-3-5	4
Scheme 5	1-2-3-4	5

**Table 6:** Comparison of the final classification results of different data-processing methods.

Dataset	Random	Scheme 1	Scheme 2	Scheme 3	Scheme 4	Scheme 5
Average accuracy of raw feature data	88.38%	81.15%	85.38%	84.23%	83.85%	83.46%
Maximum accuracy of PCA	92.02%	71.54%	70.38%	75.77%	74.62%	62.31%
RFE-LightGBM highest accuracy	94.84%	94.23%	93.08%	95.00%	93.46%	94.23%

## Data Availability

Data are contained within the article.
